# Promoting Less Complex and More Honest Price Negotiations in the Online Used Car Market with Authenticated Data

**DOI:** 10.1007/s10726-021-09773-8

**Published:** 2022-02-17

**Authors:** Andreas Engelmann, Ingrid Bauer, Mateusz Dolata, Michael Nadig, Gerhard Schwabe

**Affiliations:** Department of Informatics, University of Zurich, Binzmühlestrasse 14, CH-8050 Zürich, Switzerland

**Keywords:** Information asymmetry, Dishonesty, Negotiation behavior, Authenticated data, Used car market, P2P online negotiations/sales

## Abstract

Online peer-to-peer (P2P) sales of used and or high-value goods are gaining more and more relevance today. However, since potential buyers cannot physically examine the product quality during online sales, information asymmetries and consequently uncertainty and mistrust that already exist in offline sales are exacerbated in online markets. Authenticated data platforms have been proposed to solve these problems by providing authenticated data about the negotiation object, integrating it into text-based channels secured by IT. Yet, we know little about the dynamics of online negotiations today and the impact of the introduction of authenticated data on online negotiation behaviors. We address this research gap based on two experimental studies along with the example of online used car trade. We analyze users’ communicative and strategic actions in current P2P chat-based negotiations and examine how the introduction of authenticated data affects these behaviors using a conceptional model derived from literature. Our results show that authenticated data can promote less complex negotiation processes and more honest communication behavior between buyers and sellers. Further, the results indicate that chats with the availability of authenticated data can positively impact markets with information asymmetries. These insights provide valuable contributions for academics interested in the dynamics of online negotiations and the effects of authenticated data in text-based online negotiations. In addition, providers of trade platforms who aim to advance their P2P sales platforms benefit by achieving a competitive advantage and a higher number of customers.

## Introduction

Not least since the Covid-19 pandemic have (re)sale platforms between peers (primarily customer to customer/C2C) gained in growing popularity (Lei et al. 2021). Text-based computer-mediated communication (CMC) such as chat facilitates the trading process, allowing the involved parties to exchange information about a product and its price (Johnson and Cooper 2015). However, online sales chats have been associated with counter-normative social behaviors. First, as online trade does not provide the possibility to examine the product quality physically, the problems of information asymmetries and consequently uncertainty and mistrust that already exist in offline sales are accelerated online (Huston and Spencer 2002; Dimoka et al. 2012). Second, online sales chats lack nonverbal communication, leading sellers to have a greater tendency to bluff, exaggerate, deceive, and lie. This dishonesty hurts the sense of trust among potential buyers and sellers (van der Toorn et al. 2014), obstructs the information exchange, and consequentially reduces the negotiations’ success rate (Naquin and Paulson 2003). Finally, to avoid disadvantageous deals, buyers tend to hedge the risk of adverse transactions into the negotiation, leading in many cases to protracted and complex discussions with high costs for the involved parties (Akerlof 1970). In general, the greater the set of possible outcomes, the greater the complexity of the negotiation as it increases the transaction space (i.e., the space of possible offers and counteroffers that buyers and sellers take into account) (Susarla et al. 2010; Susarla 2012). The complexity in negotiations and the fear of dishonesty and deception deter buyers and sellers from entering and using the platforms and eventually reduce the number of closed deals on these platforms. Targeting these negotiation problems is both a challenge and an opportunity for trade platform providers.

Authenticated data platforms address information imbalances and dishonesty in online negotiations and their consequences (Zavolokina et al. 2020). These proposed solutions build on the promises of distributed ledger technologies that ensure data immutability and integrity (Cho et al. 2021) and provide proof of provenance of the collected data (Koutroumpis et al. 2020). Such novel platforms could make available authenticated data about the history of the negotiation object (Notheisen et al. 2017), integrating them into text-based channels secured by IT (Söllner et al. 2012). The integration could potentially counteract dishonesty and deception, promote information exchange, and lead to better negotiation outcomes by changing the participants’ negotiation behaviors. However, little has been done to understand the dynamics of online negotiations and the effects of the introduction of authenticated data in negotiation contexts, and their impacts on online negotiation behaviors.

This exploratory study examines this research gap along with the example of Switzerland’s online peer-to-peer (P2P) used car market, which is intensely characterized by information asymmetry and dishonesty (Lee 1998; Lee et al. 1999). Particularly prominent here are dishonesties regarding a used vehicle’s mileage or accident history, with lies very often being told to suggest a better condition of the car. This false information is hard to identify for an interested party. To get a better understanding of these deceptive practices, we first seek to understand the as-is situation, asking:

RQ1: Which behaviors do participants engage in during online P2P negotiations in the used car market?

Since research has proposed that authenticated data availability likely decreases information asymmetry in P2P online used car markets, we ask how this may change the negotiation dynamics:

RQ2: How do participants adapt their P2P online negotiation behaviors when accessing authenticated data about an offering?

To answer those questions, we conducted two experimental market games in the context of the online used car market, exploring current negotiation behaviors in P2P used car sales and the impact of authenticated data on these negotiation behaviors. We used a taxonomy of strategic moves and turns for our behavior analysis, making it possible to compare specific classified sequences of actions in negotiation processes. The analysis includes the consideration of communication between negotiators and the implementation of negotiation strategies (Thompson 1990) and allowed us to identify changes in negotiation behavior between the treatments. The first experimental study served to understand the differences in negotiation behaviors between the current negotiation behavior in a conventional market and those where authenticated data is available. The second experimental study served the purpose of gaining a more in-depth understanding of the negotiation behavior following an observed shift in dishonest behavior as evasive behavior when authenticated data are available. Thus, it included only an experiment with authenticated data.

The findings provide rich insights into buyers’ and sellers’ current negotiation behaviors and strategies during the trade of used cars. Besides that, this study shows that authenticated data can change seller and buyer behaviors to clearer and less complex negotiation processes, promote honesty, and resolve trust issues to some extent. These findings are relevant for both research and practice. Researchers can better understand the dynamics of text-based P2P negotiations by gaining more detailed insights into the communicative and strategic actions involved in price negotiations and authenticated data’s influences on such behavior. Practitioners, especially platform providers, can learn how users use authenticated data in text-based media and how a platform can support negotiators more to extend their own business and acquire new platform users.

## Related Work

### Challenges in Established Online P2P Sales Negotiations

Online sales between private individuals (i.e., P2P sales) have become an important field in e-commerce. In P2P sales, the parties negotiate prices using online communication such as private chats or instant messaging (IM) (Johnson and Cooper 2015). Research into CMC clarifies that the dynamics of such conversations depend on the features offered by the medium (Bødker and Andersen 2005; Johnson and Cooper 2015; Kurtzberg et al. 2018). Further, the design of the features may strongly impact lying behaviors (Hancock et al. 2004). Due to specific visualizations or input/output modes, CMC technologies can enhance information exchange and disclose deception (Hancock et al. 2004), which is important for effective negotiation. If one party in an economic transaction has more or better information than another party, they can use it to their advantage (Christozov et al. 2006). Thus, the dyadic context is characterized by competitive behavior where dishonesty and deception are usual ways to abuse information advantages and overcome uncertainty (Carlson et al. 2004; Gaspar et al. 2019). The more is known about the other party’s goals, baseline price, motives, or trust situation, the more beneficial the agreed outcome is likely to be for one (Lewicki et al. 2016). However, honest behavior in the form of truthful disclosure increases negotiators’ willingness to share information and make concessions (Paese et al. 2003). Currently, there is little insight into what happens during private buyer–seller communication. Most importantly, we lack an understanding of the dynamics of online negotiation, especially practices used in price negotiations via text-based channels such as chat or IM (Johnson and Cooper 2015), even though this P2P sales type is flourishing.

The online used car market is strongly affected by information asymmetry, as described by Akerlof (1970) for the used car market generally. Buyers struggle to evaluate a car’s value, while sellers usually have more experience with it and know its strengths and weaknesses better than buyers do. Thus, buyers of a car can only approximate its true quality based on previous experiences or comparing similar offers and fall behind in negotiations with uncertainty and mistrust. These challenges make the used car market an appropriate area of investigation to understand the dynamics of buyer–seller negotiation behaviors (Paese et al. 2003; Hofstede et al. 2019) and measures to overcome information asymmetry. For instance, while it is usually vital to withhold certain information from a prospective buyer (e.g., an accident), it may be essential to disclose information (e.g., whether the car is accident-free). Some of the exchanged information may be accurate (e.g., whether it is a nonsmoking car is easy to verify), and some of it may be presented in a way that the buyer believes certain things that are more advantageous to oneself (e.g., the indication of a careful driving style that suggests a good condition).

Although the problems caused by information asymmetries have been studied extensively in the literature, little is known about their impacts on text-based P2P negotiations, how to counteract them, and how information exchange can be promoted. Thus, to understand how negotiators behave and which tactics they use, we will consider the research on the nature of P2P negotiations.

### Online P2P Communication Behaviors and Strategic Actions

Negotiations involve at least two parties with different—sometimes diametrical—preferences or priorities (Lewicki 2016). They aim at agreements that can be integrative (win–win) or distributive (win-lose) (Thompson 1990). In integrative negotiations, the interests of the negotiating parties are not purely competitive, and usually, multiple issues are involved (Galinsky et al. 2016). The negotiators try to achieve greater utility by identifying and prioritizing additional value, benefits, and resources to reach the best possible outcome for all parties involved, also known as a variable-sum game (Thompson 1990). In distributive negotiations, one party tries to achieve a goal that conflicts with the other party’s intention. The parties are assumed to conflict regarding allocating a particular number of resources from usually a single issue to attain the greatest possible benefit (Galinsky et al. 2016). One party’s gain is the other party’s loss. Gains and losses add up to zero (if personal preferences are ignored), also known as a zero-sum game. Characteristically, negotiations involve both integrative (creating value) and distributive (claiming value) strategies that negotiators must combine and adapt to achieve mutually beneficial solutions (Olekalns and Weingart 2008). However, negotiations over the price of an object, in which sellers typically prefer higher and buyers typically prefer lower agreement prices, tend to be more distributive, and they focus on one single issue—price determination (Johnson and Cooper 2015).

During price negotiations, the parties change their strategic actions based on the available information and response to one another (Griessmair and Gettinger 2020), making the negotiations dynamic (Olekalns and Weingart 2008). The participants’ strategic decisions are made at the start of the negotiation without information about the other party. They must adapt and change their behaviors overtime to counteract the other party’s actions (Olekalns and Weingart 2008). Thus, bargaining consists of sequential combinations of tactical actions and redirections (Weingart and Olekalns 2004) that include opening offers, concession types, and the use of threats and commitments (Kolb 2004). These interaction sequences can be captured and analyzed to understand behavior, its adaptation, and change. On the one hand, the sequences consist of several tactical actions, which Kolb (2004) calls a framework of *moves* and *turns* that describes critical moments in a negotiation. For instance, a threat is a move intended to force the other party to reach an agreement (“This is my final offer, take it or leave it.”). On the other hand, additional strategic actions are relevant in sales negotiations. For instance, negotiators should be aware of possible alternatives, such as the *best alternative to a negotiated agreement* (BATNA): for a buyer seeking a new offer respectively another seller, for a seller switching to another prospective buyer (Lewicki et al. 2016). Such actions can influence the course of a negotiation.

Thus, negotiators can use moves, turns, and actions such as BATNA to introduce dishonesty, deception, and distrust into a negotiation because the counterpart cannot easily verify it for accuracy. How negotiators behave in this context has been studied for offline negotiations. But how these dynamics are established in online negotiations has not yet been studied in-depth. Thus, we will answer RQ1 by arguing that sequence types and dishonest behaviors can be identified in negotiation processes to describe negotiators’ behaviors in online price negotiations.

Further, the sequence types render changes visible when the preconditions of negotiations are changed. We investigate how these changes occur, for instance, when new negotiation elements are introduced using the promising approach of authenticated data.

### Authenticated Data as an Enabler for Trust and Information Exchange

A novel approach to addressing the challenges of information asymmetries and mistrust in the used car market that has recently been proposed in research is the provision of *authenticated data* (Hammi et al. 2018)—sometimes also referred to as *certified data* (Chanson et al. 2019) or *trusted data* (Notheisen et al. 2017; Bauer et al. 2019). It has been proposed that distributed ledger technology can effectively ensure high-quality, authenticated data: It can help ensure the immutability of data and provide proof of provenance (Koutroumpis et al. 2020). It also provides technical means to support the integrity of data provided by multiple parties (Notheisen et al. 2017) and data collected by sensors (Chanson et al. 2019). Finally, blockchain allows one to manage the ownership of digital assets (i.e., authenticated data) in a decentralized way (Miscione et al. 2020). Hence, authenticated data provided by blockchain are not easy to copy or reproduce, as is the case with non-authenticated data. If data entries were copied and distributed outside the system, they would lose their reliability and value. So, blockchain authentication can verify ownership of a vehicle’s digital representation. Thus, in the context of the used car market and other P2P sales, a distributed ledger can help address information asymmetry problems by supporting trust in authenticated histories of the sales objects (Stahl and Strausz 2017; Zavolokina et al. 2020).

Some propose that a distributed ledger may induce a change in the trust relationship of buyers, who currently have a strong need for interpersonal trust rather than a need for trust in the data quality (Notheisen et al. 2017). Others have proposed useful design guidelines for establishing trust in such data platforms (Zavolokina et al. 2019). However, how this plays out is still unknown.

While the studies offer promising results about the potentials of authenticated car data to reduce information asymmetries in the used car market (Bauer et al. 2019), it is unclear how authenticated data will be used in this context and how they may change established negotiation behaviors. Further, how the availability of authenticated data may affect the sellers’ tendency to lie about a car’s quality and whether they will change behaviors not related to the content of the data itself, such as BATNA, remain open questions. We claim that we can answer RQ2 by identifying behavior changes and showing what consequences for platform providers and individuals result from them.

### Impact of Authenticated Data on Negotiations

Authenticated data can exercise significant impact on how participants behave during negotiations. Their availability makes it possible to rely on credible and correct information during the conversation, at least regarding facts or figures covered by the authenticated data. In negotiations without authenticated data, participants need to collect reliable information on their own while referring to trusted sources outside of the negotiation context. Even if such information enters the conversation, the reliability of the source can be questioned by one of the parties or contrary data might be provided. This is different if the authenticated data is delivered directly during the negotiation by an intermediary or a platform trusted by both participants. The role of the authenticated data increases even further if the disclosed information is based on facts and is revealed appropriately, accurately, and is unbiased, so participants can trust and rely on it (Yu et al. 2021). These characteristics enable the availability of more correct information with source credibility that increases information adoption, information usefulness, and information credibility (Ismagilova et al. 2020), especially if available in a low-complex, predefined, and structured manner. Overall, we presuppose that providing access to authenticated data during negotiation will increase the supply and use of correct and credible information in the negotiation conversations. This will have an impact on honesty and complexity of the conversations.

As lying, bluffing, exaggerating, manipulating, or concealing are common behaviors in negotiations (Lewicki and Polin 2013) honesty is essential to build a beneficial relationship between the negotiating parties. The antecedent of honesty in the context of this study is the negotiator’s decision to reveal information truthfully and candidly (Citera et al. 2005) and the verifiability of the disclosed information details that a negotiator reveals to appear credible (Palena et al. 2021). Thus, supporting a truthful and credible disclosure of verifiable data may increase conversational honesty.

Further, conversations between buyers and sellers have a certain complexity because information asymmetry increases the set of possible outcomes (i.e., width of the buyers' and sellers' transaction space) and consequentially buyers tend to incorporate the risk of disadvantageous transactions into the deal. Often, it is a sequence of utterances in which the focus is on obtaining and releasing information and which, in their entirety, represent the process of negotiation an minimize the transaction space. Usually, it is difficult to predict how the decision process will proceed as it varies in its degree of risk and uncertainty (Laubert and Geiger 2018). A conversation is complex when there are many different actions to choose from (options), and the negotiator does not know which move/turn will cause which reaction (Gibbs and van Orden 2012). A conversation is less complex when this is more predictable, i.e., the conversation patterns are simpler (fewer options). In other words, complexity is inversely correlated with the uncertainty about the structure and outcomes of a conversation as it reduces the width of the transaction space (Susarla et al. 2010; Susarla 2012). In the negotiation context, the conversational complexity influences the decision of how to react to tactical actions (i.e., which move/turn do I take as a negotiator or how do I respond with a move/turn to the action of my counterpart?). From this, a reduction of conversation patterns (and their inherent relationships) in price negotiations can reduce conversational complexity. Authenticated data can have a corresponding influence here since its predefined character and its correctness and credibility allow only specific reactions (e.g., a prospect will not ask further about the truthfulness of an authenticated piece of information after receiving it).

**Fig. 1:**
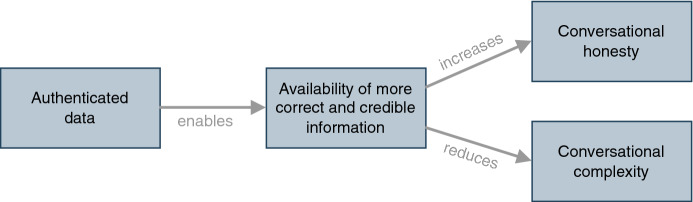
Conceptional model

In conclusion, we developed the following conceptual model for our study, which we will use throughout our elaborations to investigate the influences of authenticated data. Figure 1 depicts the positive relation of the availability of more correct and credible information and conversational honesty, as well as the negative relation between the availability and the conversational complexity. It also shows that authenticated data functions as an enabler for the increased availability of correct and credible information.

## Data Collection and Analysis

### The CarMarket Game

This study was conducted in the context of a larger design science research project (Peffers et al. 2007) called Cardossier, a real-world project in which several car-related companies are collaborating to develop a consortial distributed car ledger. In collaboration with representatives from the Cardossier project, we developed a blockchain-based IS artifact—CarCerti. CarCerti simulates a blockchain-based information system that enables a multitude of stakeholders to collect and manage all relevant events during the life cycle of a car. CarCerti, therefore, provides trusted car history data, such as general car data (e.g., factory configurations) and usage-specific data (e.g., mileage history), as authenticated data.

Since we explore current negotiation behaviors and changes therein through the availability of authenticated data, we conducted two experimental studies using a market simulation game—the CarMarket game—in 2018 and 2020. Besides validating theoretical models, experimental techniques are a well-suited method to discover and describe phenomena and their correlations in the course of exploratory research (Stebbins 2001). While there are critics of laboratory experiments (Levitt and List 2009), other studies have shown that lab experiments’ findings can be a successful method for exploring and evaluating ISs’ effects (Hashim et al. 2017).

**Fig. 2:**
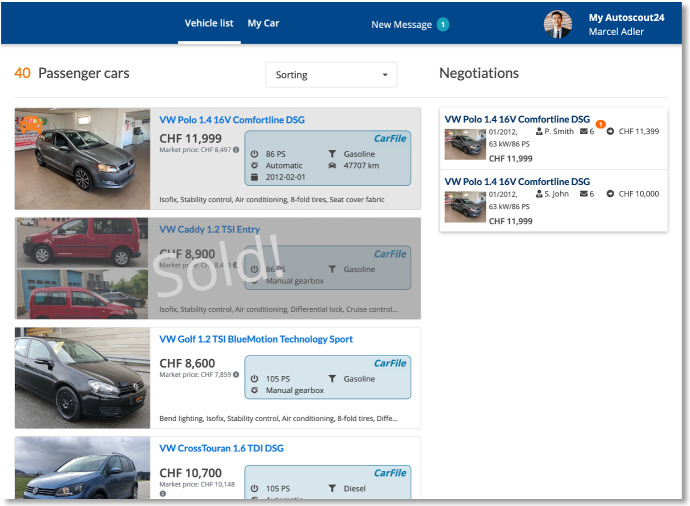
Overview over the CarCerti marketplace and negotiations (from the 2020 study)

**Fig. 3:**
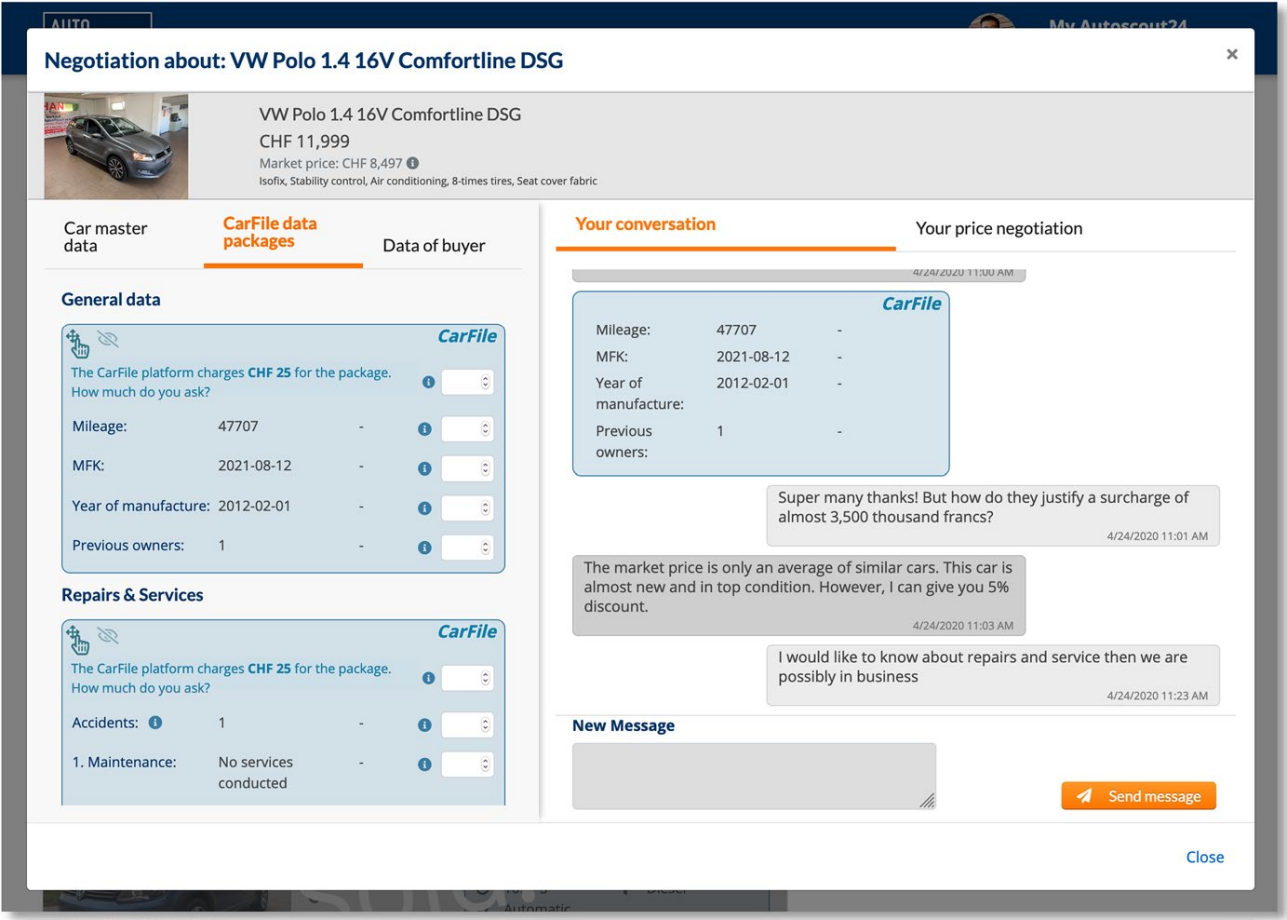
The message and offer dialogue (from the 2020 study)

The CarMarket game, which we developed for this study, simulated the used car market and allowed us to embed authenticated data. It provides core functions copied mainly from an existing used car platform (Fig. 2). New additions to the CarMarket game, such as the chat function or the integration of authenticated car data, were designed and discussed with experts from the field (Fig. 3).

The figures show the interface used in experiment 2 (2020) from the user’s perspective. We only slightly improved the user interface based on the feedback collected during experiment 1 (e.g., authenticated data was integrated directly into the chat dialogue, making it easier for the test persons to concentrate on the negotiation process than switching between information sources). CarCerti features are highlighted in blue. The marketplace provides the cars on offer and the negotiations a buyer/seller is involved in.

The negotiation dialogue shows an ongoing negotiation. The left half of the negotiation dialogue contains data from CarCerti; the right half is a chat negotiation. Notably, CarCerti data can be directly embedded into the chat.

### The Experimental Setup and the Data Collection

After smaller pretests, we conducted our final experiments in June 2018 and April 2020, each with a group of students. Using students as study objects can yield results as reliable as actual customers if they have sufficient knowledge about the study environment (Höst et al. 2000). To account for enough background knowledge, all the participating students were first introduced to the online used car market and the CarCerti project (a week in advance, preparation material in the form of a written document, and a screencast with a walkthrough; early in the lecture, a verbal explanation, and a live walkthrough). These included a description of the general procedure and goals as well as an explanation of the game’s key features.

For the initial experiment (2018), the goal was to understand the differences in negotiation behaviors between conventional and CarCerti treatments. Fifty students from an advanced Bachelor’s class in IS participated. They were incentivized to act as realistically as possible by the possibility of earning bonus points for their final exam and the chance to win various vouchers. The objective for each player was to maximize their relative revenues. We chose the relative revenue as a measure of success to account for the fact that the cars’ values differed slightly. We randomly assigned the students to predefined user accounts and roles (i.e., buyer or seller) used for both treatments (conventional and CarCerti-supported). Every player participated in each game round in this experimental setting using the same role, following a between-subject design (Charness et al. 2012). After the game, 47 of 50 participants voluntarily agreed to be interviewed in a co-located setting. The interviews contained questions on their negotiation behaviors. The interview guide was created according to Myers and Newman (2007). All interviews were semi-structured and were recorded and transcribed verbatim, according to Saldana (2015). Most lasted between 30 and 40 min.

The second study in 2020 aimed to gain a more in-depth understanding of the negotiation behaviors using authenticated data from CarCerti. Accordingly, we identified a set of focal phenomena for an in-depth exploration in the first experiment. Since the phenomena were related to the impact of CarCerti, only the CarCerti treatment was conducted in a second experiment. We used a concatenated exploration approach to expand on the findings of our first study in this subsequent study (Stebbins 2001). This procedure provides a chance to substantiate descriptions of focal phenomena and the relationships between them and potentially improves the generalizability of the findings (Stebbins 2006). In the second experiment, 72 students from the Bachelor IS class agreed to participate, and the introduction and incentive system remained unchanged. Only the between-subject design setting was changed to a within-subject one, and the roles were switched in round 2, such that each student enacted both roles throughout the experiment and could reflect on it afterward. After the game, 13 participants were interviewed in MS Teams (owing to Covid-19 restrictions). The interviews contained more detailed questions about their negotiation strategies and behaviors. Most lasted between 30 and 40 min. Again, the interviews were recorded and transcribed.

Further, all chat communications, CarCerti data transactions, and car transactions from both experiments were available and analyzed.

### Data Analysis

All qualitative interview data were coded using an open coding process. The analysis process had two steps. First, a graduate student conducted the collected material’s initial coding in a bottom-up approach to identify the most salient themes and patterns. Based on this, two researchers deductively and inductively processed the core themes to understand their relationships (axial coding). This process generated insights into negotiation behaviors and strategies (Saldana 2015). The coding schema concentrated on intentions, communicative actions, and strategic actions applied by the participants. The interviews revealed the participants’ perspectives on their behaviors and assumptions about other subjects’ behaviors.

The messages of all chat histories were broken down and abstractly coded into strategic moves and turns according to Kolb (2004) (proposed as a framework) by two researchers in an interactive process to avoid contradictory interpretations. Two senior researchers supervised the coding process, and precedent cases were discussed in a team. Analyzing the so-coded chat histories allowed us to identify sequence patterns as the participants’ dominant negotiation behavior, which were comparable between the different treatments. For the *moves* used for the analysis, see Table 1. To facilitate understanding, we provide an example for each move from our results.

**Table 1:** Description of the most common strategic moves [according to Kolb (2004)]

Move	Description	Example
Challenging competence or expertise	Questioning the other party’s claims of experience and competence	What justifies the price of 12,500 when the market price is only 8,400?
Demeaning ideas	Attacking the idea, leaving little room for the counterpart to respond, even if the idea is reasonable	Your offer is an insult.
Criticizing style	Casting a counterpart as an irrational person who cannot be reasoned with because they are perceived to be overreacting or inconsiderate	The market value is only 9,300. Make me a good offer.
Making threats	Attempting to force a choice from the other party or to corner them	Final offer, otherwise I’m afraid I’ll have to look elsewhere.
Appealing for sympathy or flattery	Attempting to silence the counterpart to make it harder for the other person to achieve their own goals	How much of a discount would you offer a good buyer?:-)

These moves can be responded to with reactive countermoves or with turns. *Countermoves* are moves like the ones above but provided as a response. *Turns* are ways in which negotiators can challenge or respond to a move in a restorative way to get out of a defensive position (e.g., distract from the actual move) or in a participatory manner to get the other person to cooperate (e.g., revealing information) (Kolb 2004). Turns were coded according to Kolb’s framework to analyze the negotiation sequences. Table 2 provides the descriptions and examples of the turns.

**Table 2:** Descriptions of the turns [according to Kolb (2004)]

Turn	Description	Example
Interruption	Disrupting a move by a short pause in the action. It can help a negotiator to regain control, for instance	(Since participants could have multiple negotiations simultaneously, we ignored the time periods between messages in our analysis.)
Naming	Naming a move signals recognition that you know what is happening and suggests that you will not be fooled	If you had [CarCerti], you would not offer me 12,500.
Questioning	Questioning a move shows that something about it is not understood. It is thrown back at the other person to imply that the negotiator is unsure what prompted it	And not even a warranty on the engine. How can I be sure?
Correcting	Correcting a move with an improving turn substitutes a motivation or different version implied by the move and can neutralize the move	Please look at the full analysis! The car has some added value.
Diverting	A redirecting turn directs the focus to the problem. It is a way to ignore the implication of the move and for the negotiator to take control (e.g., distract from the negotiation object’s condition)	What would your final price be if I were to purchase the data?
		But has a very up-to-date MFK.

Besides the moves and turns, the coders looked for other patterns across the dataset. They identified (1) the use of BATNA as a tactical action in the negotiation process and (2) the reference to CarCerti arguments in game rounds with CarCerti. The latter was used to analyze authenticated data’s influences on the participants’ strategic behaviors and to identify how references to CarCerti changed the sequence of turns and moves in the subsequent dialogue.

The analysis of the collected and coded material was iterative. The results were based on a quantitative evaluation of the frequencies and a qualitative examination of the content of negotiation actions and the interview data. Thus, first, we counted the occurrences of the moves, turns, BATNA, and references to CarCerti to identify the most frequent ones. These insights enabled us to understand quantitative differences between the treatments. Second, we counted the sequences of actions (which turn followed which move, and vice versa) and aggregated them into patterns. This allowed us to create an abstract model of negotiation behaviors and internal dependencies between the expression types and compare them across the treatments. Third, we checked the messages for dishonesty and deception, i.e., we compared the data of the vehicles in the game to corresponding statements in the chats, thereby determining when lying or deception was taking place.

For the analysis, negotiations that contained only numerical offers and counteroffers without further information exchanges were not narrowly examined. Finally, for the conventional round of experiment 1 (E1 = the conventional round), 78 conversations with 293 text messages were analyzed. Accordingly, round 2 with CarCerti (E1 = the CarCerti round) consisted of 75 conversations with 323 messages, and the sole round of experiment 2 (E2 = the CarCerti round) provided 323 conversations with 1,696 messages. In total, we analyzed 476 negotiations containing 2,312 messages. The upcoming section describes this in some detail.

## Results

In the following, the results of the experiments are presented, which were based on the coded text messages that buyers and sellers had sent one another during the used car negotiations. The subsections will first examine the engagement of the negotiators in the conventional treatment and then the adaptation of their behaviors after the introduction of CarCerti data. Each of the two subsections will highlight the negotiators’ behaviors from three perspectives: (1) the intensity of use, (2) sequential order, and (3) dishonesty and deception. The intensity of use focuses on the frequency with which tactics are used, regardless of when they are used. Because it aggregates over time, this level of analysis can provide insight into the dominant tactical actions of negotiators. The sequential order perspective respects the temporal component. It can capture patterns of strategic aggregation (sequence types) that can be used to identify changes of conversational complexity between negotiations. The dishonesty and deception perspective seeks to expose the deceptive and dishonest behavior regarding information practiced by the negotiators. It identifies when true and when false statements are made and aims to identify changes in conversational honesty. These perspectives describe the characteristics of the participants’ behaviors during P2P online negotiations in a multidimensional way, thereby answering RQ1 and identifying differences between the conventional and the authenticated-data treatment in many dimensions, contributing to RQ2.

### Which Behaviors do Participants Engage in During P2P Online Negotiations?

The results showed that negotiation participants show more engagement in specific actions than in others: the more frequently an action was used, the more engaged they were in pursuing the corresponding purpose.

#### Observations Based on Most Uses

While the buyers used negotiation *moves* (e.g., to initiate negotiation sequences), the sellers primarily used negotiation *turns* (i.e., they responded to buyers’ negotiation prompts). The buyers most often used *challenging competence or expertise*, *appealing for sympathy or flattery*, with the other moves used less regularly. The sellers primarily used the turns *correcting* and *diverting*, and the move *appealing for sympathy or flattery.* Table 3 summarizes the results and provides evidence that the moves and turns were often used differently, revealing the most prominent actions owing to their frequency.

**Table 3:** The uses of moves, turns, and BATNA in E1 (the conventional round; 50 participants)

Move	Frequency			Most prominent owing to frequency^a^
	Buyer	Seller	Total	
Challenging competence or expertise	24	–	24	x
Appealing for sympathy or flattery	15	9	24	x
Making threats	10	2	12	
Criticizing style	2	1	3	
Demeaning ideas	1	–	1	
*Turn*				
Correcting	2	61	63	x
Diverting	3	6	9	x
Questioning	2	–	2	
Naming	–	2	2	
Interruption	–	–	–	
*Other actions*				
BATNA	3	6	9	x

^a^The move *making threats* was often used owing to the time pressure (the limited time of the experiment), which represents a bias. Thus, we omitted it from our considerations

Overall, based on counting the buyers’ and sellers’ actions in the conventional treatment, we identified that *challenging competence or expertise* and *appealing for sympathy or flattery* were the most frequent moves. Combined, they accounted for 75% of all moves observed in the conversations (48 of 64). The move *challenging competence or expertise* was used only by buyers while *appealing for sympathy or flattery* was used by both parties (buyers: 62.5%). We observed *making threats* only 12 times and the remaining moves even less frequently, which is why we did not analyze them further. Interestingly, buyers were the ones who used moves most (52 of 64), while the sellers most often responded to them with turns (69 of 76 turns coded in the data). By far, the most frequent turn was *correcting*, with an overall frequency of 63, of which 61 cases were associated with sellers. There were nine instances of observations of *BATNA*.

#### Observations Based on the Sequential Order

To characterize the interactions between the various prominent actions and to compare temporal structures, we referred to the sequential structure of the observed negotiations: (1) by counting how often various actions followed one another in sequential order and (2) by attending to the messages’ content to understand their interrelationships. We noted that the negotiation moves and turns occurred in sequential patterns. We identified three patterns, which we will now describe.

The *challenge and withstand pattern*: Normally, the buyers saw an attractive advert and offered a price they were willing to pay for the car. In 11% of cases, they directly used *challenging competence or expertise* on the seller by confronting information in the advert or their price estimation with some evidence. In more than 50% of the cases, the sellers reacted to this through *correcting* or *diverting*. Specifically, they tried to neutralize the challenge by providing additional evidence or pointing to information possibly ignored by the buyer (*correcting*), or they went off-topic, for instance, by referring to personal experiences (*diverting*). Both responses empowered the seller to resist the claim. In all other cases, the sellers simply responded by making an alternative offer (frequently without any additional comment, merely a number), did not react to *challenging competence or expertise* at all, or even aborted the chat right after this challenge by the buyer.

The *gain closeness* pattern: If a buyer used *appealing for sympathy or flattery* at some point in the dialogue, the most frequent response was *appealing for sympathy or flattery* by the seller in about 44% of the cases. However, in 21% of the cases, sellers responded by *correcting*. Qualitative insights into the chat data showed this behavior when creating a personal closeness to encourage the other party to indulge.

The *friendly enforce* pattern: If buyers or sellers responded to *appealing for sympathy or flattery* simply by saying that they are also involved in other negotiations (*BATNA*), they sought to force a decision in a friendly way.

**Fig. 4:**
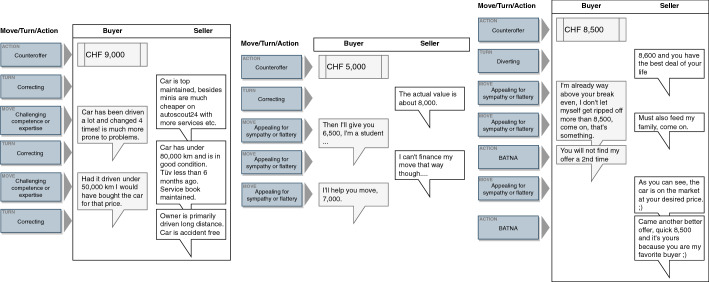
Excerpts from conventional chat histories representing sequence types

These sequential patterns showed the most prominent behavior during the negotiations. Figure 4 shows exemplary chat histories that map these patterns. Based on these striking sequential patterns, we can detect changes in the further course of the study.

#### Behavior in Terms of (Dis)Honesty

A contextual analysis enabled us to check the participants’ truthfulness during negotiations. The chat messages revealed the contextual use of dishonest behaviors and deception. The *correcting* turn (i.e., mostly specification of vehicle data) and *BATNA* turned out to be the actions with the largest potential for lies because the comparison with the current facts in the game data often showed invalidity. The sellers used the *correcting* turn to correct a buyer’s claim regarding the car’s condition. From 51 identified data-related statements connected to this turn, 47% were lies. Further, there were seven false price statements (deception about the price in the advert). Similar dishonest behaviors could be seen when buyers or sellers used BATNA. More than 55% of the cases were untrue, and the counterpart did not realize this. Table 4 summarizes the frequencies of occurrence.

**Table 4:** The relationships between honesty and dishonesty in E1 (the conventional round; 50 participants) by frequency

Content of the turn correcting	Turns affected	Statements (1-n per turn)	
		Buyer	Seller
False data statement	13	–	24
Truthful data statement	21	–	27
False price statement	7	–	7
*BATNA*			
Lied (no such alternative)		2	3
Truth told (existing alternative)		1	3

We also found confirmation for these insincere behaviors in the interviews. The interviewees stated that the seller’s perceived honesty was fairly low because the buyers could not be sure if the data presented by the sellers were (un)truthful. Further, some sellers stated that they had lied about the data during the negotiation. For instance:

[…] the seller could easily lie to you if only he knows the value. If the buyer does not know the data, he quickly pays too high a price. […]

[…] the first time I sold, I had a vehicle that had been in an accident. My goal was to get rid of it at the market price, and I cheated a bit and was able to sell it for that.

[…] Because the buyers did not really have a way to trust the sellers, because there were not like other platforms such reviews or so. You just had to say something, and then that’s just right or wrong.

#### The Interpretation of the Observed Behaviors

**Fig. 5:**
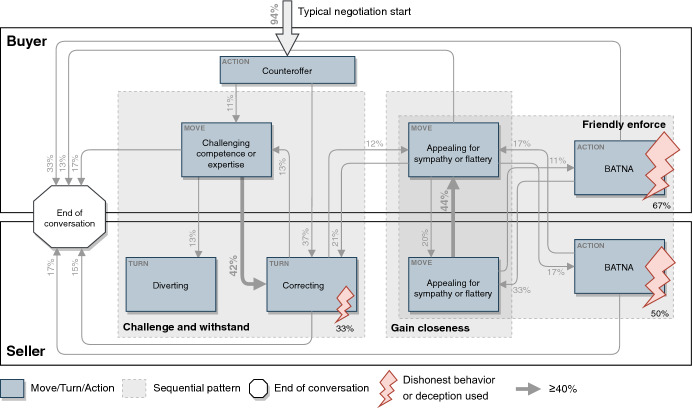
Model of negotiation behaviors in the conventional online used car market

From the sequences of moves, turns, BATNA, and insights into dishonest behaviors and deception, we could develop the following model, which depicts the dominant used car negotiation behavior used. The cumulative data at the arrowheads show the relative frequency of the corresponding response. We only used sequences that impacted more than 10% (thus, not all elements have an incoming and an outgoing arrow). A stronger arrow highlights reactions with a frequency greater than 40%. Figure 5 shows the resulting model, visualizes the sequences, which we identified as sequence types, and shows the utilized dishonest behaviors relating to the moves, turns, and BATNA.

Based on this conventionally used car market model, we can compare behavioral changes as authenticated data enters the market.

### How do Participants Adapt their P2P Online Negotiation Behaviors When Accessing Authenticated Data About an Offering?

To answer RQ2, it was necessary to observe differences in the treatment with CarCerti data compared to the conventional one. Therefore, we analyzed the data of E1 (the CarCerti round) in the same way to reveal diversities.

#### Observations Based on the Dominant Use

In addition to the actions in the conventional treatment, the use of authenticated data in the form of *CarCerti arguments* (a reference to or information from CarCerti) yielded insights into such actions’ effects. The buyers engaged in the same negotiation moves as in the conventional treatment. Still, this time, they also used authenticated data (e.g., to support their argumentation). The sellers again primarily used negotiation turns. The buyers most frequently used *challenging competence or expertise*, *appealing for sympathy or flattery*, *CarCerti arguments*, and less often than other moves such as *making threats*, *criticizing style*, and *demeaning ideas.* The sellers primarily used the turns *correcting* and *diverting,* and this time, less frequently in the move *appealing for sympathy or flattery.*

The move *challenging competence or expertise* was again used exclusively by buyers (n = 44). In contrast to the conventional game round, it was used 83% more often this time, a significant increase. The move *appealing for sympathy or flattery* was again used second most often (n = 26) and more often by buyers (n = 17). Since we observed *making threats* only 11 times and the remaining moves even less frequently, we did not consider them further. The *correcting* turn was used most frequently again (n = 70) but now exclusively by the sellers. The *diverting* turn was again used the second most often (n = 34), but in contrast to the conventional round with a significant increase in use (278%) and this time significantly more frequently by buyers (n = 23). Since the other turns*—questioning*, *naming*, and *interruption*—were used very seldom or not at all, we did not investigate them further. A *BATNA* argument was used 11 times, but now almost exclusively by the sellers (only one buyer). Additionally, a CarCerti argument was used 80 times. In sum, in this CarCerti round, the same negotiation actions were as prominent as in the conventional round, with some significant differences in the frequency of use.

#### Observations Based on the Sequential Order

A detailed quantitative analysis of the interactions in this round again disclosed sequences of negotiation actions. We observed a change of the sequential patterns from the conventional round and a new pattern.

The *severe challenge and withstand* pattern: The move *challenging competence or expertise* was now used by the buyers with an additional *CarCerti argument* in more than 54% of the move usages. In almost 60% of this move’s uses, sellers responded with the turn *correcting* (45.5%) or *diverting* (13.6%). Thus, it was somewhat more common (about 10% more frequently) to respond with these turns when CarCerti was available. It seems more challenging for the seller to resist the move *challenging competence or expertise* because the claims from CarCerti were harder to refute. Further, we observed that the sellers also used a *CarCerti argument* when responding: 12 times with the turn *correcting* and once with the turn *diverting.* This seems to indicate a change in the negotiation behavior. In 12 cases (27.7%), there was no response from the seller.^1^ In all other cases, the negotiation was aborted (n = 2; 4.5%), or another argument was used (n = 4; 9.1%).

The *missing counterargument* pattern: The buyers now responded very often with the *diverting* turn to the sellers’ *correcting* turn (33%). The buyers did not respond appropriately when the sellers had used CarCerti data. Here, the counterargument seems to be missing, which enabled the buyers to counter the sellers’ claim.

The disappearance of the *gain closeness* pattern: The move *appealing for sympathy or flattery* was used almost as often by buyers and sellers compared to the conventional round. However, this move was no longer responded to with the same move, *appealing for sympathy or flattery*, so the cycle from the sequential pattern *gain closeness* from the conventional round no longer existed. It was usually responded to with another move or turn.

The disappearance of the *friendly enforce* pattern: A *BATNA* with the move *appealing for sympathy or flattery* was hardly used so that the pattern *friendly enforce* was not identified either. Further, the turn *correcting* was used only twice as a response, and a *BATNA* argument only once. In all other cases, the negotiation was aborted (n = 10; 38.5%), or another reaction was used (n = 9; 34.6%).

**Fig. 6:**
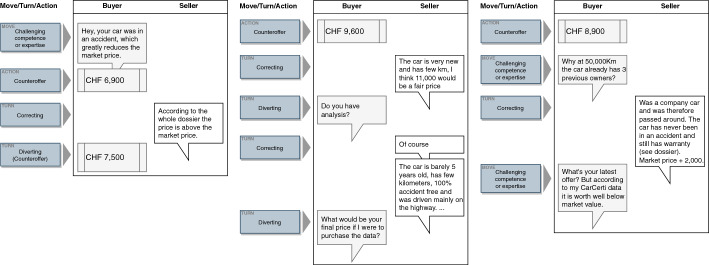
Excerpts from CarCerti game chat histories representing sequence types and dishonesty change

The following excerpts from several CarCerti chat histories illustrate patterns of the change in negotiation behaviors (Fig. 6). The left-hand history shows a typical changed negotiation starting with the move *challenging competence or expertise* using *CarCerti arguments*, and subsequently the *correcting* turn; the middle one the changed behavior in using a *diverting* turn after a *CarCerti argument* by the seller; and the right-hand history the change in dishonest behavior, which we will now explain in some detail.

#### Behaviors in Terms of (Dis)Honesty

The correcting turn was again of interest in analyzing the participants’ dishonest behaviors in the CarCerti treatment. In the chat histories, we found 49 data-related statements in this turn, but this time only 10% lied. The reduction of lies is a significant decrease (37%) compared to the conventional treatment (a *change to truthful data*). On the other hand, we found a significant increase in deception (by 185%) concerning the car’s price (*a change to price deception*). A *BATNA* argument was used 11 times (4 times true, 7 times lied) and almost exclusively by the sellers (only one buyer). *BATNA* use seems to have shifted to the sellers and was used as an evasive behavior for further deception (*change to evasive behavior*). Table 5 provides an overview of these relationships.

**Table 5:** The relationships between honesty and dishonesty in E1 (the CarCerti round; 50 participants) by frequency

Turn correcting	Turns affected	Statements (1-n per turn)	
		Buyer	Seller
False data statement	5	–	5
Truthful data statement	27	–	44
False price statement	20	–	20
*BATNA*			
Lied (no such alternative)		–	7
Truth told (existing alternative)		1	3

The data indicates that sellers can no longer use false data statements owing to the introduction of authenticated data. So, they used evasive behavior by shifting dishonesty to misstatements that were harder to refute, such as untrue pricing and lying *BATNA*. To further explore those relationships, we conducted another experiment consisting of only one CarCerti treatment to gain deeper insights into dishonest behaviors in a market with authenticated data, as explained in 3.2. We prepared the results of.

E2 (the CarCerti round) in the same way as before but omitted the usage overview and concentrated on the analysis of dishonesty. Table 6 shows the results of this analysis.

**Table 6:** The relationships between honesty and dishonesty in E2 (the CarCerti round; 72 participants) by frequency

Turn correcting	Turns affected	Statements (1-n per turn)	
		Buyer	Seller
False data statement	6	–	10
Truthful data statement	69	–	90
False price statement	8	–	8
*BATNA*			
Lied (no such alternative)		–	24
Truth told (existing alternative)		–	14

Concerning *change to truthful data*, only 10% of the used data arguments relating to the *correcting* turn were lies. This aligns with observations from the first experiment: the use of lied data has significantly decreased due to the introduction of authenticated data. Further, we saw an appreciable reduction in false price statements compared to E1 (the CarCerti round). Accordingly, the shift from false data statement to false price statement as observed in the earlier experiment could not be replicated. Concerning *BATNA*, the sellers used all BATNA, and 63% were lies. This aligns with the observations from the first experiment.

For the change in dishonest behavior, we also found confirmation in the interviews. There were frequent reports of less lying in the games with CarCerti because both negotiators can buy the data, i.e., the games with CarCerti were generally perceived as fairer:

At first, I just wanted to convince the other person that I have my CarCerti data and that everything was very good, but then you see that someone bought the file, and you can no longer lie. It was already fairer with the CarCerti and much better regulated. You could no longer cheat.

[…] and the second [car, with CarCerti] that was actually fair. I sold that at exactly the market price plus what came out of the dossier analysis.

I’m telling you the truth because you have the same information I have. So, get the information if you don’t believe me.

[…] you can understand it much better, and then you feel you’re paying a fairer price than when you don’t have that. In round two [with CarCerti], I’ve been willing to pay more if you know what you can have.

#### The Interpretation of the Observed Behaviors

**Fig. 7:**
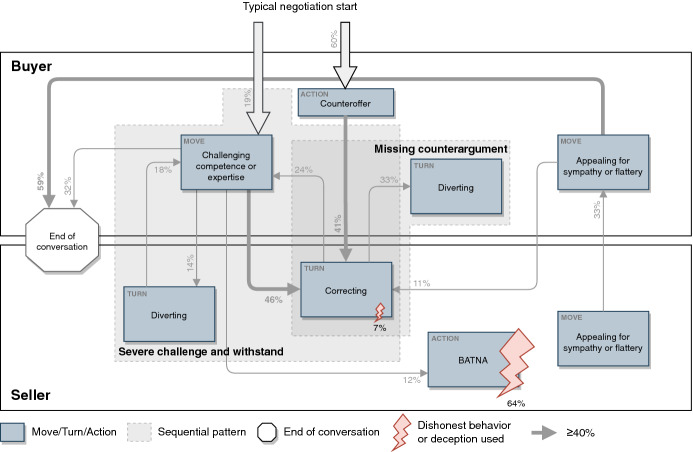
Model of negotiation behaviors in the online used car market with authenticated data (i.e., CarCerti)

From these results, it was possible to deduce how participants’ behaviors changed as a result of the introduction of CarCerti. We incorporated these changes into our model and will answer RQ2. Figure 7 shows the resulting model and visualizes this treatment’s sequence types. For the market with CarCerti, the patterns *gain closeness* and *friendly enforce* were no longer present. However, a new sequence type could be identified (*missing counterargument*), which shows buyers’ increased use of the turn *diverting.* Further, the changed dishonest behavior is illustrated.

We will now discuss these results according to the literature.

## Discussion

Not least since the Covid-19 pandemic, online negotiations have become more relevant as ever-increasing numbers of new and specially used products are being sold via the Internet. The research has identified counter normative behaviors in CMC (van der Toorn et al. 2014), as well as problems of product quality and uncertainty, which are considered amplified in online markets (Lee 1998; Lee et al. 1999). Our study has indicated that buyers and sellers, and providers will benefit from including authenticated data in their platforms.

### P2P Online Negotiations in the Online Used Car Market

We asked (RQ1): *Which behaviors do participants engage in during online P2P negotiations in the used car market?* The buyers most often engaged in challenging the sellers’ competence or expertise, while the sellers often corrected and sorted out buyers’ assertions. Also, both sides appealed for sympathy or flattery while trying to establish or strengthen interpersonal bonds. Aggressive moves were omitted, which indicates that the parties were exploring the potential for a bargain without trying to enforce it. Further, it suggests that the negotiations were about matching the price, the object, and the parties’ expectations or possibilities.

Regarding the temporal structure of negotiations, we observed several sequential patterns with inherent relationships in the traditional used car market, indicating a rather high conversational complexity. We could identify three dominant patterns. *Challenge and withstand* describes a buyer's behavior who, despite correcting or diverting turns from the seller, carried on with their original criticism. The data indicated that this pattern emerges in the early phases of the negotiation. We claim that the buyer tries to establish a strong negotiation position before making a concession regarding, for instance, the price. *Gain closeness* describes the behavior of the parties where flattery is responded to with a similar move. This allows the parties to strengthen the mutual social connection and commitment during a progressing negotiation after clarifying the initial positions. Finally, *friendly enforce* occurred in a later negotiation phase when one party uses lengthy, possibly pointless exchanges, and the other party wants to reach a clear conclusion. In this case, the other party may use BATNA to enhance the pressure on their negotiation partner. While the literature was concerned with which moves and turns occur in negotiations (Kolb 2004) our analysis unveiled typical patterns describing the sequential interdependencies between the moves and turns. The identification of typical patterns may contribute to recognizing the state of the negotiation and the conversational complexity. The conversational complexity can be visualized by the model of negotiation behaviors in the traditional used car market (Fig. 5) that shows the patterns and their relationships. We observed complex relationships between the intensively used tactical negotiation actions (high number of arrows), indicating that participants chose quasi-randomly between many different actions (Gibbs and van Orden 2012). These complex relationships make it difficult to predict what action to expect next in the negotiation process (Laubert and Geiger 2018).

Regarding deception, the results indicate little conversational honesty (cf. Gaspar et al. 2019). Although the parties did not resort to force and aggression to agree, the sellers simply lied to buyers to reach a favorable outcome—another type of immoral behavior. The interviewed participants made it clear that this was primarily a deliberate, conscious action, suggesting that people generally consider some sort of deceit to be acceptable in online price negotiations. Thus, the intuition that trusting the seller of a car is a bad idea is fairly accurate. It adds to the effect described by Akerlof (1970) by confirming that dishonesty is an inherent part of selling a used car.

However, as illustrated by the most recent results, authenticated data may offer a way to tackle this problem. We asked *how do participants adapt their P2P online negotiation behaviors when accessing authenticated data about an offering.* The major movements remained the same, confirming the overall trends identified in the conventional settings: buyers engage in challenging the sellers, who respond by correcting or diverting, and both parties try to establish social bonds by flattery. We also did not observe significant changes concerning aggressive or forceful behavior. However, the data indicated the enhanced use of diverting by buyers. And, most prominently, we could identify a new move that we refer to as the *CarCerti argument.* This move indicates how easily users adopt new information provided during the negotiation into their negotiation behavior and use it to support their argumentation. The high uptake of this information confirms that information is the fundamental resource in negotiation (Lewicki et al. 2016). Being able to refer to it gives the parties new tools to engage in a more intense conversation.

The temporal and sequential analysis of the moves and turns revealed changes of the sequential patterns and their relationships and therefore a change of conversational complexity. First, we observed a specialization of the pattern *challenge and withstand* toward the *severe challenge and withstand* pattern. The availability of additional information provides the buyer, who otherwise would have to refer to their intuition or the overall market situation, with clear-cut arguments about a car. It is foreseeable that buyers will use a CarCerti argument in connection with the challenging move of this changed pattern. Second, *missing counterargument* is a new pattern describing behavior that occurs when a buyer challenges a seller by referring to the authenticated data (i.e., a CarCerti argument), and the buyer then reacts by shifting the topic. It also explains why buyers engaged in *diverting* more than in the conventional setting. It also shows the power of authenticated data as an argument that leaves the buyer no other option than to move away from the original aspect they challenged. This movement is predictable to a certain extent. Third, the patterns *gain closeness* and *friendly enforce* disappeared which reduces the relationships between the prominent tactical actions and therefore the conversational complexity. Thus, the revealed changes provide a better prediction of the negotiators’ behavior compared to conversations under conditions without authenticated data. Moreover, the reduction of patterns and relationships shows that the presence of authenticated data can reduce the number of options considered reasonable, and therefore conversational complexity (Gibbs and van Orden 2012). The changed patterns and reduced relationships (number of arrows) are recognizable in Fig. 7. Specifically, the observations confirm the positive relation between the authenticated data, the increased availability of correct and credible information in the conversation, and the reduction of conversational complexity as stated in our conceptual model (Fig. 1).

We can identify three essential shifts concerning conversational honesty: *change to truthful data*, *change to price deception,* and *change to evasive behavior. Change to truthful data* reflects the reduction of information asymmetry about a car. The sellers can no longer lie about a car’s condition because it is possible that the buyer has seen or can see the authenticated data and that a lie about this would show the seller to be untrustworthy. *Change to price deception* refers to sellers’ disproportionate inflation of a car’s price compared to the CarCerti data. We claim this behavior occurs because the sellers can no longer present a car more positively with false data. Hence, they choose to use the price to signify the car’s quality and hope that buyers won’t use CarCerti for comparison. Finally, *change to evasive behavior* refers to the increased use among sellers of *BATNA* lies. Given more information about a car, buyers may tend to engage in lengthy negotiations to reach a better outcome. Still, sellers may wish to complete a negotiation early before all potentially risky facts about a car are discussed. Overall, the identified dishonesty patterns showed that dishonest behaviors remain, but they shift in the goal—the lies were not about the car but other aspects. While this again suggests that a certain level of deceit is accepted in online negotiations, the presence of authenticated data gives more power to the buyer, who—if they wish to—may detect some dishonesty and may effectively counteract it. Thus, we can conclude that authenticated data impacts conversational honesty. Yet, the observations show a more complex picture than we assumed in our conceptual model (Fig. 1). Specifically, the participants are more honest about technical facts and figures covered by the authenticated data, but they continue to lie about other aspects, such as the status of their parallel negotiations (BATNA). Thus, rather than a reduction of dishonesty, we observe a shift between various types of deception.

The findings support previous research on peer-to-peer negotiations in various contexts. Despite the clear incentives oriented at the price of the car, many participants positioned other issues as relevant for the negotiation, like sympathy or expertise. They were discussing them as part of the negotiation. It became part of the negotiation to present and convince the other party of one’s own expertise. This created a sort of multi-issue negotiations, which was earlier shown as supporting win–win outcomes as opposite to zero-sum outcomes (Galinsky et al. 2016). For instance, one party received the acknowledgment of their expertise while the other could benefit from a lower price. Interestingly, the accessibility of authenticated data did not change such behaviors: aspects of sympathy and expertise were still coming on the table, yet the data could be used to express or confirm one’s own expertise. Following Hofstede et al. (2019) one can confirm that a negotiation is about more than pure economic rationality. Even in an online game environment, the players try to maintain positive relations. One can interpret some cases of BATNA as an attempt to suspend unproductive negotiations without affronting the other party. This is despite the clear economic incentive structure and the risk of potentially losing the chance to reach a good deal in a parallel negotiation by spending so much time. Nevertheless, we confirm that the revelation of trusted and accurate information, even if this does not occur via the negotiator themselves as in previous research (Citera et al. 2005; Yu et al. 2021) but through a third, independent party, increases fact-related honesty and reliability of negotiation conversations. We claim that those findings hold for peer-to-peer negotiations in typical high-value transactions like in the used car market (Paese et al. 2003; Hofstede et al. 2019) or for example also in the real estate market (Galinsky et al. 2016).

### Implications for Design and Behavior Adoption

This study has contributed to the negotiation literature by identifying which behaviors emerge in online, text-based P2P sales. The results suggest that participants in an online car market are driven by profit and do not refrain from deceit to reach the desired outcome. Thus, effective ways to reduce cheating are highly valued and could improve the interaction quality in online platforms—not only in used car markets but also in freelancer hiring platforms such as Upwork or online markets features on social media such as Facebook, where the participants also negotiate prices via text-based messaging. Although these platforms verify personal data or basic information concerning the property to guarantee basic trustfulness or provide comments concerning previous transactions, the de facto negotiations and deals rely on chat-based exchanges. By including authenticated data, they could enhance the value of the channels they offer and reduce the risk of deception, improving the users’ trust in a platform. Further, by decreasing conversational complexity, they can attract more users. Since many people dislike complex negotiations and consequentially tend to avoid them, introducing authenticated data to reduce negotiation complexity can provide a promising approach for platform providers to increase their attractiveness.

However, the consequences for platforms are even more far-reaching. In particular, the introduction of authenticated data can lead to people also negotiating high-value goods via online P2P platforms. The number of customers of the platforms can increase further. In addition, customers will have more choice, as they will no longer be geographically bound, for example, but will be able to buy their high-value goods without being present at the product. Up to now, they have often still been geographically bound because they want to look at and feel the good (e.g., a car) as a product to touch. Studies of these developments will become increasingly important.

The analysis revealed that, in many aspects, chat-based negotiations resemble face-to-face ones. In both, deceit and lies play a key role (Kolb 2004; Lewicki et al. 2016; Korobkin 2020). They include moves such as *challenging competence or expertise* or *appealing for sympathy or flattery* and rely on information asymmetry and a specific amount of mistrust. However, there were differences. For instance, the analyzed chats had almost no emotionally loaded moves such as *demeaning ideas*, *criticizing style*, and *making threats.* A naive interpretation would be that online negotiations are less heated than face-to-face ones.

Overall, comparing the treatments indicated that manipulating the communication channel by introducing authenticated data strongly impacted the participants’ behaviors. This aligns well with the literature on communication channels’ impacts on communication characteristics (Bødker and Andersen 2005; Johnson and Cooper 2015; Kurtzberg et al. 2018). However, while the literature has focused on comparing various communication or media types, we have indicated that particular technology features change communication even if the medium remains the same.

Conventional P2P markets are strongly affected by information asymmetry (Akerlof 1970) and distrust between the parties; both aspects were also identified in the CarMarket game. A lack of information by one person about an offering offers great potential for the informed party to be dishonest and untrustworthy. Although measures against information asymmetry exist (Valley et al. 1998; Dimoka et al. 2012; Blundell et al. 2019), these are not appropriate in online negotiations. We have shown that providing authenticated data makes participants more honest about a car’s features and possibly reduces the necessity for interpersonal trust between the negotiating parties. Despite intense research, the mechanisms involved in establishing trust in these settings remain underexplored (Naquin and Paulson 2003; Söllner et al. 2012). We have proposed a way to, at least to some extent, circumvent the issue of trust toward the seller by suggesting how it can be replaced by trust toward the data and the platform. The availability of authenticated data may even enhance trust toward the seller: By being able to confirm their claims about a car with authenticated data, sellers could present themselves as more trustworthy. This increase in trustworthiness calls for further research concerning the trust mechanisms involving humans, machines, and data.

## Conclusions and Limitations

We have shown how the introduction of authenticated data leads to significantly clearer and less complex negotiation processes and a relocation of trust relationships and lying behaviors between buyers and sellers. These insights are particularly interesting for platform providers because it enables them to understand better how their users are using their platforms today. These insights also provide sufficient grounds for authenticated data platform providers to develop and monetize such since they show significant positive market acceptance and use. Further, we have indicated that chats enhanced with authenticated data can positively impact markets with information asymmetries, and therefore society, which currently is awash in dishonest behaviors.

Our study has limitations, which open avenues for future research. First, the experiments were conducted with students as a subject group. While many scholars have proven their use to be effective (Höst et al. 2000; Gupta et al. 2018) a classroom setting cannot reveal all facets of a real-life negotiation with thousands of dollars at stake. While our experiment’s sample size was not small, an even more significant number of participants would allow further, more in-depth statistical analysis. Especially concerning the shift in dishonest behavior, the absolute number of observed data elements of our studied phenomena is relatively small. Further investigation may provide a greater data basis here. Therefore, repeating and validating the saturation of our insights could be beneficial.

Further, there is still potential for dishonest behavior or deception. Sellers appear to engage in evasive behavior when authenticated data is available in a price negotiation. Thus, further research is needed to reduce the use of lying BATNA or make alternatives transparent (e.g., showing all other offers). The use of false price statements should also be reduced, for instance, by displaying a possible price range. However, we trust that our study will motivate researchers to continue and extend our work on negotiations supported by authenticated data and that they will analyze the integration and the effects of regulatory aspects.

## Data Availability

The data that support the findings of this study are available from the corresponding author upon reasonable request.

## Footnotes

1 This includes one seller, who did not respond to any requests in the game (n = 6).
